# Specific Strains of *Escherichia coli* Are Pathogenic for the Endometrium of Cattle and Cause Pelvic Inflammatory Disease in Cattle and Mice

**DOI:** 10.1371/journal.pone.0009192

**Published:** 2010-02-12

**Authors:** I. Martin Sheldon, Andrew N. Rycroft, Belgin Dogan, Melanie Craven, John J. Bromfield, Alyssa Chandler, Mark H. Roberts, Sian B. Price, Robert O. Gilbert, Kenneth W. Simpson

**Affiliations:** 1 Institute of Life Science, School of Medicine, Swansea University, Singleton Park, Swansea, United Kingdom; 2 Royal Veterinary College, Hatfield, Hertfordshire, United Kingdom; 3 Department of Clinical Sciences, College of Veterinary Medicine, Cornell University, Ithaca, New York, United States of America; Louisiana State University, United States of America

## Abstract

**Background:**

*Escherichia coli* are widespread in the environment and pathogenic strains cause diseases of mucosal surfaces including the female genital tract. Pelvic inflammatory disease (PID; metritis) or endometritis affects ∼40% of cattle after parturition. We tested the expectation that multiple genetically diverse *E. coli* from the environment opportunistically contaminate the uterine lumen after parturition to establish PID.

**Methodology/Principal Findings:**

Distinct clonal groups of *E. coli* were identified by Random Amplification of Polymorphic DNA (RAPD) and Multilocus sequence typing (MLST) from animals with uterine disease and these differed from known diarrhoeic or extra-intestinal pathogenic *E. coli*. The endometrial pathogenic *E. coli* (EnPEC) were more adherent and invasive for endometrial epithelial and stromal cells, compared with *E. coli* isolated from the uterus of clinically unaffected animals. The endometrial epithelial and stromal cells produced more prostaglandin E_2_ and interleukin-8 in response to lipopolysaccharide (LPS) purified from EnPEC compared with non-pathogenic *E. coli*. The EnPEC or their LPS also caused PID when infused into the uterus of mice with accumulation of neutrophils and macrophages in the endometrium. Infusion of EnPEC was only associated with bacterial invasion of the endometrium and myometrium. Despite their ability to invade cultured cells, elicit host cell responses and establish PID, EnPEC lacked sixteen genes commonly associated with adhesion and invasion by enteric or extraintestinal pathogenic *E. coli*, though the ferric yersiniabactin uptake gene (*fyuA*) was present in PID-associated EnPEC. Endometrial epithelial or stromal cells from wild type but not Toll-like receptor 4 (TLR4) null mice secreted prostaglandin E_2_ and chemokine (C-X-C motif) ligand 1 (CXCL1) in response to LPS from EnPEC, highlighting the key role of LPS in PID.

**Conclusions/Significance:**

The implication arising from the discovery of EnPEC is that development of treatments or vaccines for PID should focus specifically on EnPEC and not other strains of *E. coli*.

## Introduction

Ascending infection of the upper female genital tract with Gram-negative bacteria causes pelvic inflammatory disease (PID) or endometritis in women, with the influx of neutrophils and macrophages leading to accumulation of pus in the uterine lumen [1]. Female genital tract infections with Gram-negative bacteria are also an important cause of infertility, pre-term labour and chronic pelvic pain [2]. Bacteria such as *Chlamydia trachomatis* are well adapted to colonise the human endometrium and disease models have been established in mice using infusion of bacteria [3]. Alternatively, pathogen-associated molecules such as lipopolysaccharide can be used *in vivo* to establish PID in mice [4], [5]. Ascending infection of the female genital tract with a wide range of bacteria occurs in almost all cattle after parturition [6], [7]. This infection often leads to disease of the upper female genital tract, which can be called pelvic inflammatory disease or metritis [8]. Indeed, about 40% of animals develop PID within a week of parturition, and ∼20% have endometritis that persists for >3 weeks [9].

Infection of the endometrium with Gram-negative *Escherichia coli* is the first step in the disease process for developing PID in cattle, preceding infection by the other bacteria such as *Arcanobacterium pyogenes* [6], [10]. The presence of *E. coli* is associated with the acute phase protein response, the severity of PID and the extent of the infertility [6], [7], [10]. There is a wide genetic diversity of *E. coli* in the environment and feces [11], [12]. So, the widely held assumption was that these genetically diverse fecal *E. coli* randomly and opportunistically contaminate the endometrium to cause PID. However, there are well characterised pathogenic strains of diarrheagenic *E. coli* (DEC) and extra-intestinal pathogenic *E. coli* (ExPEC) such as uropathogenic *E. coli* (UPEC) that infect tissues other than the endometrium [13]–[15]. So, in addition to the expectation that multiple random environmental strains of *E. coli* cause PID, or that DEC or ExPEC could be involved, there remains the possibility that the endometrium is infected by previously un-identified strains of *E. coli* that are pathoadapted to cause PID. Essential pathogenicity traits of *E. coli* include adhesion to epithelial cells [14], motility mediated by flagella (identified by the H serogroup) [13], and toxins such as shigatoxin, heat stable and labile toxins, and lipopolysaccharide (LPS, identified by the O serogroup) [16]. Host cells recognize LPS via a specific receptor complex comprising of Toll-like receptor 4 (TLR4), CD14 and MD-2, which leads to an inflammatory response including the secretion of cytokines and chemokines [17]. Endometrial epithelial and stromal cells also express the TLR4 complex and LPS stimulates secretion of chemokines such as interleukin-8 (IL-8) and disrupts endocrine function by switching prostaglandin secretion to predominantly prostaglandin E_2_ [18], [19].

The present study tested the hypothesis that PID is associated with distinct strains of *E. coli* that are pathogenic for the endometrium. Animals were monitored in a longitudinal study for PID and uterine bacteria were isolated each week during the post partum period. Specific clonal phylogenetic groups of *E. coli* were associated with the presence of PID throughout the year from different animals. Bacteria associated with PID were more adherent and invasive for endometrial cells *in vitro* than *E. coli* collected from the uterus of clinically unaffected animals, and provoked the greatest inflammatory response. These bacteria also colonised the endometrium of mice *in vivo* to establish PID. Finally, the bacteria lack pathogenicity genes typically associated with virulence in *E.coli*, although they did possess the ferric yersiniabactin uptake gene (*fyuA*), and endotoxin LPS was important for stimulating the inflammatory response. Thus, specific endometrial pathogenic *E. coli*, designated as EnPEC, are adapted to the endometrium and cause PID.

## Results

### Specific Clonal Groups of *E. coli* Were Associated with PID

**Figure 1:**
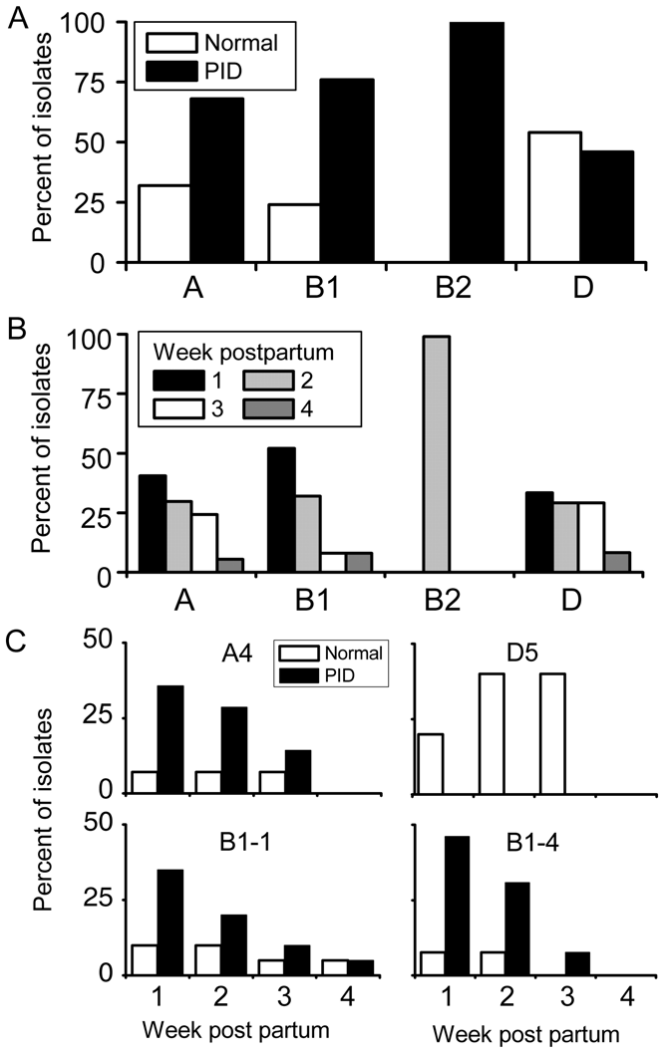
Phylogeny of *E. coli* isolated from the postpartum uterus. (A) Distribution of uterine *E. coli* isolates in phylogenetic Triplex-PCR group A (n = 37 isolates), B1 (n = 51), B2 (n = 3) and D (n = 23) between clinically unaffected animals (normal, □) and animals with pelvic inflammatory disease (PID, ▪). (B) Distribution of the uterine *E. coli* isolates in Triplex-PCR group A to D between weeks one to four post partum. (C) Distribution of bacteria in RAPD genotype A4 (n = 14), B1-1 (n = 20), B1-4 (n = 13) and D5 (n = 5) collected 1 to 4 weeks post partum between clinically unaffected (normal, □) or animals with pelvic inflammatory disease (PID, ▪). Bars represent the percent of isolates within a genotype.

Bacteria were isolated from the uterine lumen each week for 4 weeks post partum with concurrent examination for PID, using 64 Holstein animals studied for one year, as described previously [6]. The study used 114 uterine *E. coli* isolates, which were categorised by Triplex PCR into phylogenetic group A (n = 37; 32% of isolates), group B1 (n = 51; 45% of isolates), group B2 (n = 3; 3% of isolates), or group D (n = 23; 20% of isolates). There were 77 (67.5%) *E. coli* isolates from the uterus of 41 animals with PID and 37 (32.5%) isolates from 23 unaffected animals. Triplex group A or B1 bacteria were more likely to be isolated from the animals with uterine disease (Fig. 1A; P<0.05) and to be collected during the first or second week postpartum (Fig. 1B; P<0.05) than group D isolates.

The genetic diversity of the 114 uterine *E. coli* was further explored using Random Amplification of Polymorphic DNA (RAPD; Fig. S1) with ten RAPD genotypes identified within Triplex group A, 11 in group B1, 2 in group B2 and 10 in group D. However, several isolates shared the same RAPD genotype: 14/37 isolates were in RAPD genotype A4; 39/51 in B1-1, B1-2 or B1-4; and 10/23 in D4 or D5. Bacteria from these RAPD genotypes were associated with uterine disease (A4, 11/14; B1-1, 14/20; B1-2, 5/6; B1-4, 11/13) or clinically unaffected animals (D4, 3/5; D5, 5/5), and these strains were isolated from different postpartum animals over a period of 12 months. The bacteria from the A4, B1-1, and B1-4 genotypes also tended to be isolated during week 1 or 2 post partum (Fig. 1C). In addition, *E. coli* isolated 1 week after parturition from the B1-1 and B1-4 genotype were more likely than the remaining week 1 isolates to be associated with a subsequent infection by *A. pyogenes* (13/16 vs. 16/34; P<0.05). The isolates in the A4, B1-1, B1-4 and D5 RAPD genotypes were subjected to serotyping and MLST to further examine if they were clonaly related and to explore differences between the bacteria associated with PID and those collected from unaffected animals. The serotypes for these isolates are reported in SI Table S1. Although there was a wide range of serotypes, the O73:H16 serotype was frequent amongst B1-4 isolates from PID animals, and the O(84,172):H+ serotype was common to the D5 genotype.

**Figure 2:**
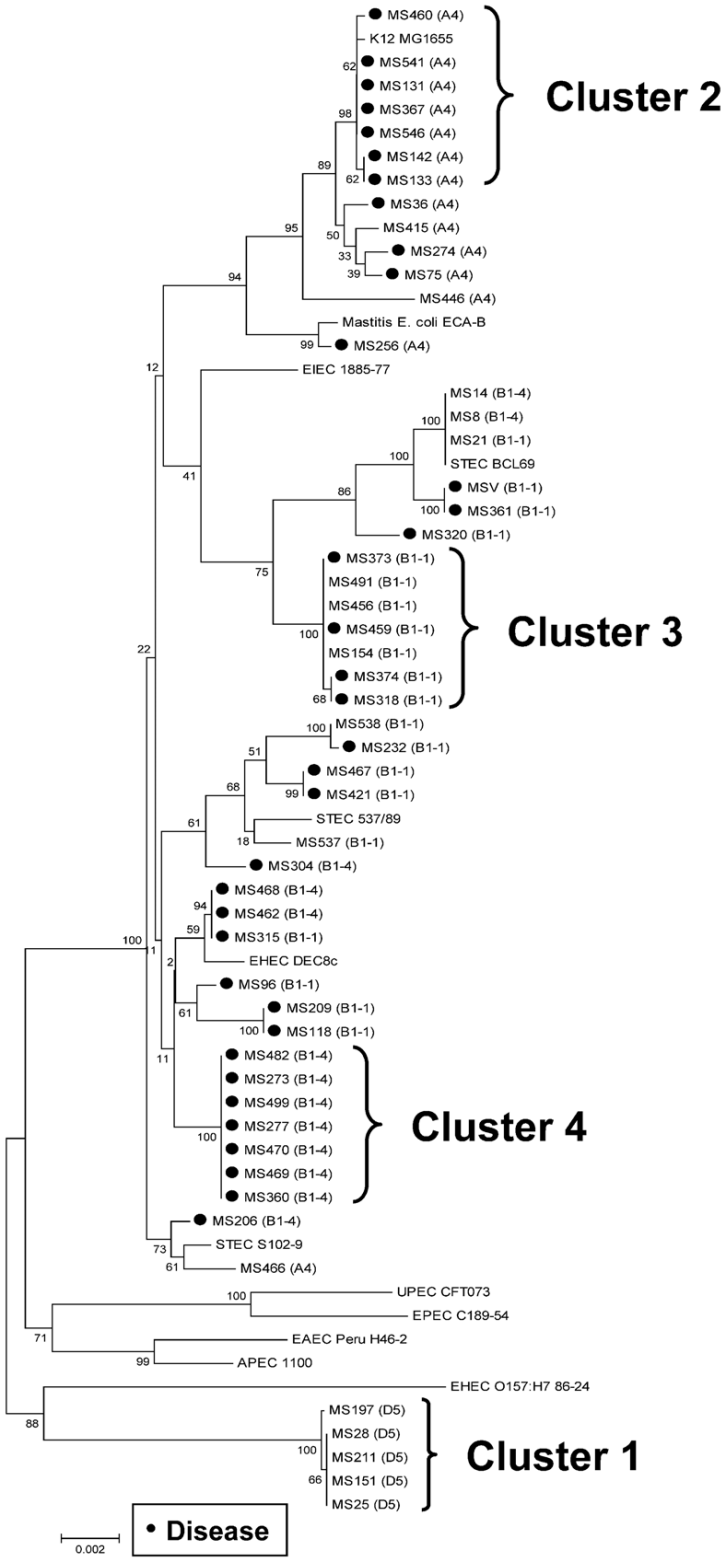
Multilocus sequence typing of *E. coli*. Dendrogram of *E. coli* isolates from the uterus of clinically unaffected or diseased (•) animals based on Multilocus sequence typing (MLST) using genes listed in Materials and Methods. The boostrap values are provide, the RAPD groups are in parenthesis and the MLST clusters of bacteria are indicated. Eleven reference strains of *E. coli* are also presented in the dendogram, including *E. Coli* K12 (K12), enteroinvasive *E. coli* (EIEC), enteropathogenic *E. coli* (EPEC), enterohaemorrhagic *E. coli* (EHEC; human or bovine origin), Shiga toxin-producing *E. coli* (STEC; bovine origin), uropathogenic *E. coli* (UPEC), avian pathogenic *E. coli* (APEC), enteroadherent *E. coli* (EAEC) and a bovine mastitis strain of *E. coli*.

Multilocus sequence typing (MLST) revealed that many of the *E. coli* isolates were from four clonal clusters (Fig. 2 and Table S1). One cluster of 5 bacteria from the D5 RAPD genotype was only isolated from clinically unaffected animals (designated as cluster 1), and all strains in this cluster were O(84,172):H+ and had novel st7 alleles and clonal groups. Other clusters of *E. coli* were associated with PID in 7/7 (cluster 2, RAPD B1-1), 4/7 (cluster 3, RAPD A4) and 7/7 animals (cluster 4, RAPD B1-4). The *E. coli* strains in clusters 2 and 3 varied in serotype and MLST st7 and clonal group. However, the strains in cluster 4 were all serotype O73:H16, st7 471 and clonal group 4 (Table S1). The *E. coli* in MLST clusters 1 to 4 lacked associations with reference strains of *E. coli* (Fig. 2), including human and bovine DEC (including O157:H7), UPEC and a bovine mastitis strain of *E. coli*, except for the non-pathogenic strain, K12. Further comparisons of the MLST data with the *EC*MLST database (Michigan State University) also found that cluster 4 bacteria were similar to a prototypical *E. coli* in clonal group 41 (TW08574). The occurrence of subsequent *A. pyogenes* infection was more common in animals that had clusters 2 and 4, than 1 or 3 *E. coli* infection (11/14 vs. 3/12; P<0.05). None of the animals had bacteria from more than one MLST cluster and the bacteria were isolated throughout the year (see Fig. S2). Having identified clonal groups of *E. coli* from the uterus of postpartum animals, subsequent experiments explored how the bacteria in MLST clusters 2 to 4, associated with PID, differed from the *E. coli* in cluster 1, which were collected from unaffected animals.

### Clonal Groups of *E. coli* Associated with PID Were Most Adherent to Endometrial Cells

**Figure 3:**
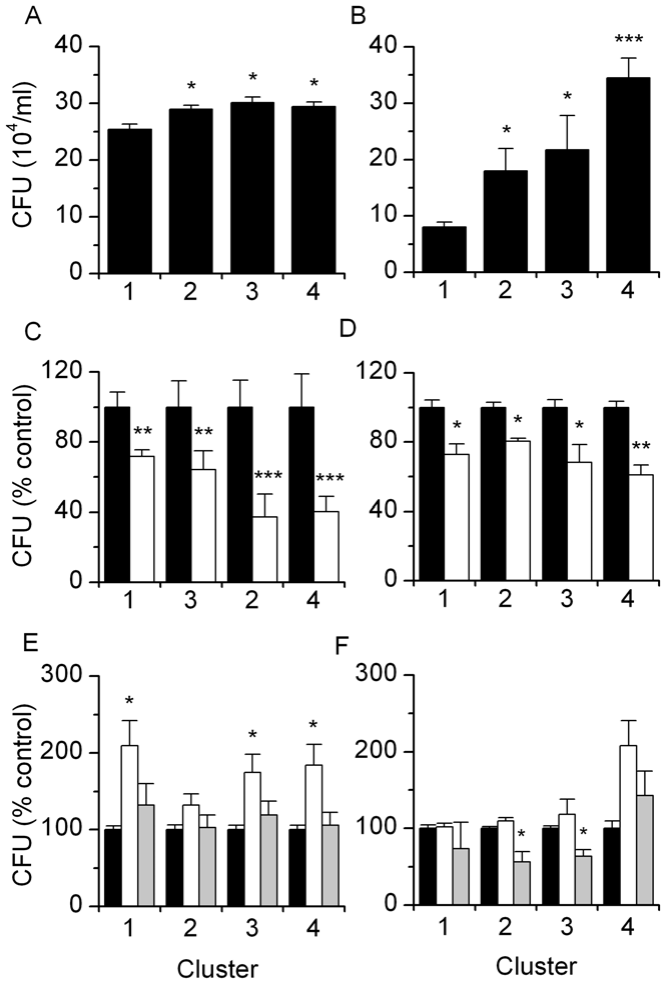
Adhesion of *E. coli* to bovine endometrial cells. Bacteria from MLST clusters 1 to 4 (n≥4 per cluster) were added to confluent (A) epithelial or (B) stromal endometrial cells, incubated for 1 h and the number of adherent CFU measured; experiments were repeated on 4 separate occasions with 4 to 6 isolates from each cluster. Values were compared to cluster 1. Adherence of bacteria to (C) epithelial or (D) stromal cells was inhibited by addition of 2.5% D-Mannose to the culture medium (□), which blocks the Type 1 fimbriae; values were compared to untreated control medium (▪), within each cluster. Adherence of the bacteria was also modulated by pre-treatment of (E) epithelial or (F) stromal cells for 48 h with medium containing 5 ng/ml progesterone (□) or 3 pg/ml estradiol (▪); values were compared to untreated control medium (▪), within each cluster. Bars represent the mean + SEM of four experiments, * P<0.05, ** P<0.01, *** P<0.001.

To evaluate the pathogenicity of *E. coli* isolated from the endometrium, the adhesion and invasion of bacterial isolates from cluster 1 (n = 4), cluster 2 (n = 5), cluster 3 (n = 4) and cluster 4 (n = 6) were measured at 10 x multiplicity of infection (M.O.I.) using primary bovine endometrial cells. Bacteria associated with PID from cluster 2, 3 or 4 were more adherent to epithelial or stromal cells than cluster 1 bacteria from clinically unaffected uteri (Fig. 3A, B). A common mechanism for adherence of *E. coli* to host mammalian cells involve Type I pili, including FimH adhesin [20]. Type 1 fimbrial adhesion was also involved in bacterial adherence to endometrial cells because addition of an inhibitor of fimbrial adhesion (2.5% D-Mannose) to the culture medium reduced the adherence of bacteria from all clusters to epithelial or stromal cells, compared with the same isolates in untreated control medium (Fig. 3C, D). However, Type 1 fimbrial adhesion did not differ between bacteria from the different MLST clusters, as determined by agglutination profiles for *Saccharomyces cerevisiae* (Fig. S3).

Endometrial cell function is regulated by ovarian steroid hormones. Uterine infection is easier to establish during the progesterone-dominated luteal than estradiol-dominated follicular phase of the ovarian cycle, and steroids regulate the endometrial immune response [19], [21], [22]. To test if ovarian steroids may affect host-pathogen interactions, endometrial cells were grown in control culture medium, or in medium containing progesterone at luteal phase concentrations or estradiol at ovarian follicular phase concentrations, for 48 h before measuring bacterial adhesion. Although the effect was not significant for all the *E. coli* MLST clusters, progesterone increased bacterial adhesion to epithelial cells (Fig. 3E) and estradiol reduced adhesion to stromal cells (Fig. 3F), compared with control medium.

### Clonal Groups of *E. coli* Associated with PID Were Most Invasive for Endometrial Cells

**Figure 4:**
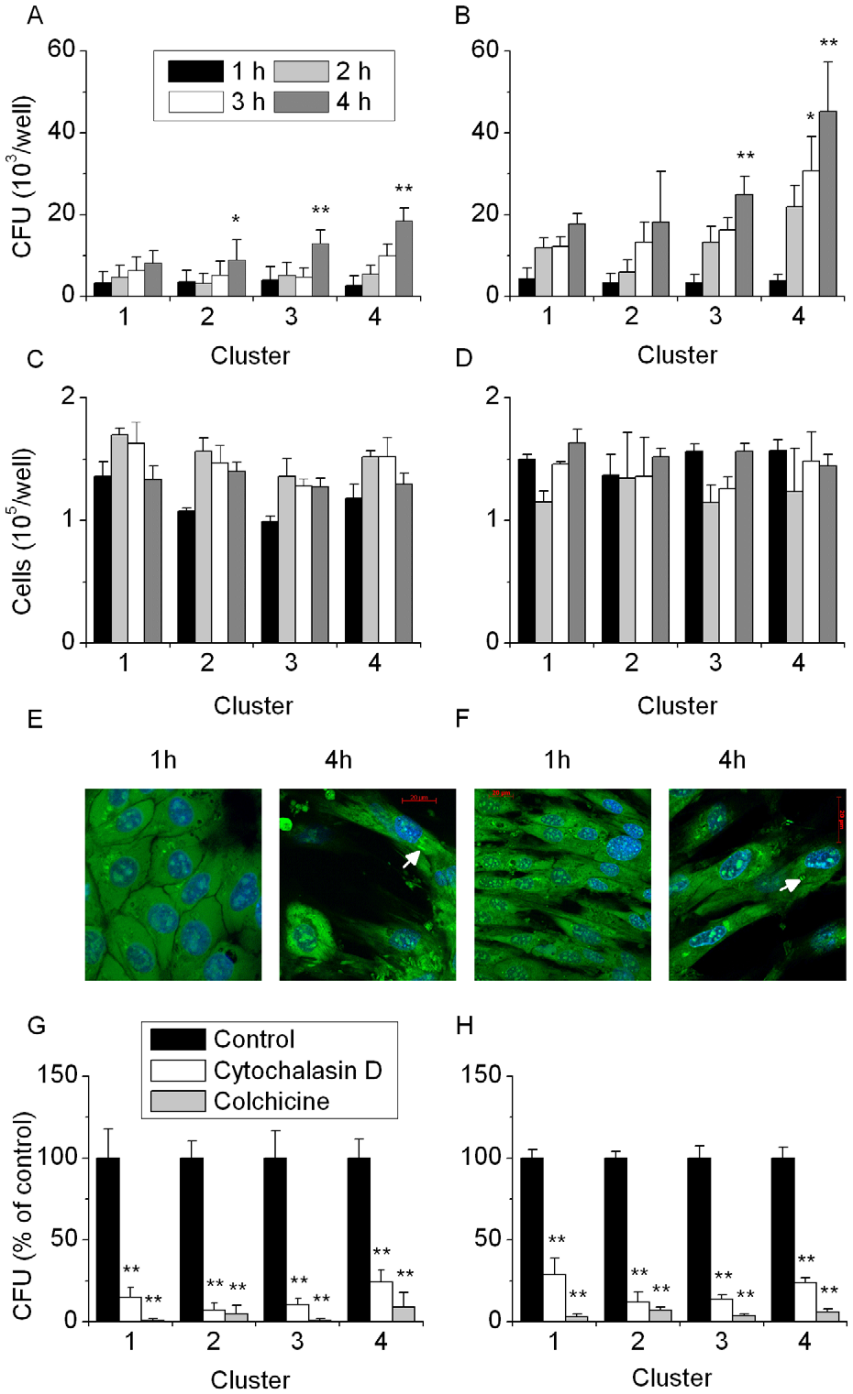
Invasion of host cells by uterine *E. coli*. Bacteria from MLST clusters 1 to 4 (n = 4 per cluster) were added to confluent (A) epithelial or (B) stromal cells, incubated for 1 to 4 h before addition of gentamicin for 2 h, and the number of adherent CFU measured; experiments were repeated on 4 separate occasions with at least 4 isolates from each cluster. Values were compared to cluster 1 at the corresponding time point. The number of (C) epithelial or (D) stromal cells present at the end of each experiment was determined. Bacteria (arrow) were visualised using the Syto 9 DNA stain in (E) epithelial and (F) stromal cells after 4 h but not after 1 h of incubation. Bacterial invasion of (G) epithelial or (H) stromal cells was inhibited by the addition of cytochalasin D (□) or colchicine (▪) to the culture media; values were compared to untreated control medium (▪), within each cluster. Bars represent mean + SEM of four experiments, * P<0.05, ** P<0.01.

Invasion of endometrial cells by *E. coli* at 10 M.O.I. for 1, 2, 3 or 4 h was tested by gentamicin protection assays [23]. Cluster 3 and 4 bacteria were more invasive than cluster 1 *E. coli* for epithelial (Fig. 4A) or stromal cells (Fig. 4B); cell survival was not affected (Fig. 4C, D). Bacteria could be seen within the cytoplasm after 4 h but not after 1 h incubation with epithelial (Fig. 4E) or stromal cells (Fig. 4F). To explore the mechanism of invasion, gentamicin protection assays were performed with endometrial cells that had been incubated for 1 h in control medium or in medium with the host cytoskeletal inhibitors cytochalasin D (microfilaments) or colchicine (microtubules) before addition of 10 M.O.I. bacteria. Cytochalasin D markedly reduced (P<0.01) the invasion of epithelial and stromal cells by *E. coli* from each cluster and invasion was almost completely blocked by colchicine (Fig. 4G, H), compared with control medium.

### LPS from the *E. coli* Associated with PID Stimulated the Greatest Inflammatory Response

**Figure 5:**
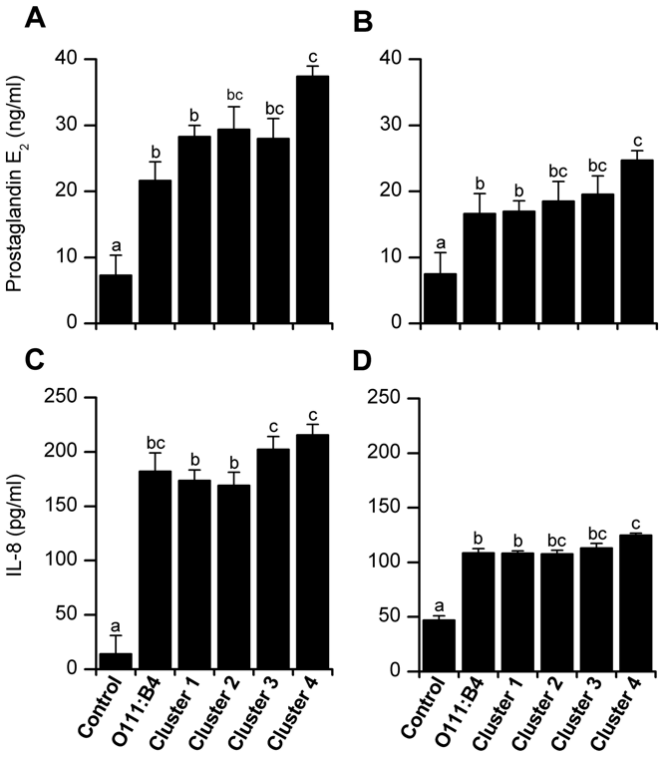
Host cell response to uterine *E. coli*. Prostaglandin E_2_ and interleukin-8 (IL-8) concentrations accumulated over 24 h in the medium of endometrial epithelial (A, C) and stromal cells (B, D) treated with LPS. Cells were treated with control media or media containing 1 µg/ml LPS purified from *E. coli* O111:B4 or from bacteria isolated from the uterus of animals and classified into MLST cluster 1 to 4 (n = 3 isolates per cluster). Prostaglandin E_2_ and IL-8 concentrations were determined by RIA and ELISA, respectively. Data are presented as the mean + SEM of at least three experiments. Values with different superscripts differ significantly, P<0.05.

To further explore the mechanism of pathogenicity, endometrial cells were treated with LPS commercially purified from *E. coli* O111:B4 (Invivogen, San Diego, CA, USA) or purified from MLST clusters 1 to 4 *E. coli* (n = 3 isolates per cluster). Prostaglandin E_2_ (PGE) and interleukin-8 (IL-8) accumulations in media were measured because LPS impacts endometrial cell endocrine and immune function to stimulate secretion of PGE [18], and the chemokine IL-8 attracts neutrophils and macrophages to the endometrium *in vivo* [24]. Addition of LPS stimulated accumulation of PGE from epithelial (P<0.001; Fig. 5A) and stromal cells (P<0.001; Fig. 5B). More PGE accumulated in the media of epithelial and stromal cells treated with LPS from bacteria in MLST cluster 4 than 1 (P<0.05). Furthermore, treatment with LPS stimulated secretion of IL-8 from epithelial (P<0.001; Fig. 5C) and stromal cells (P<0.001; Fig. 5D). More IL-8 also accumulated in the media of epithelial or stromal cells treated with LPS from bacteria in MLST cluster 4 than 1 (P<0.05).

### Uterine *E. coli* Possessed Few Genes Commonly Associated with Pathogenicity

To identify bacterial genes that may be important for establishing PID, bacteria were examined for 17 virulence genes associated with adhesion, invasion and virulence in DEC and ExPEC [14], [25], [26]. The *E. coli* isolated from PID and unaffected animals lacked the virulence genes for K99 fimbrial subunit, *E. coli* fimbrial adhesin subunit F1845, *E. coli* CS31A fimbrial subunit precursor, heat-stable toxin (*Sta*), Shiga-like toxin types 1 and 2 (*stx1* and *stx2*), cytotoxic necrotizing factors 1 and 2 (*cnf1* and *cnf2*), intimin-γ (*eae*), colicin V plasmid (colV), group II capsule (*kpsMII*), invasion of brain endothelium (*ibeA*), P fimbrial assembly units (*papC*), afimbfial adhesin (*afaB-afaC*), S fimbriae (*sfaD-sfaE*), and F1C fimbriae (*focG*) (Table S1). However, strains of *E. coli* isolated only from animals with PID and not unaffected animals, possessed the ferric yersiniabactin uptake receptor (*fyuA*) gene, which is associated with bacterial iron uptake [27].

### *E. coli* Associated with PID Were Resistant to Antimicrobials

Antimicrobials are commonly used to treat PID [9]. So, the antimicrobial sensitivity to seven common antimicrobials was examined by antimicrobial disc diffusion assays using all the available isolates where the results were unequivocal, including all the *E. coli* strains from clusters 1 to 4. Antimicrobial sensitivity patterns did not differ significantly between phylogenetic groups (Fig. S4). However, *E. coli* from MLST clusters 2, 3 and 4 were more likely to have resistance to at least one antimicrobial than cluster 1 bacteria (cluster 1, 0/5; cluster 2, 2/7; cluster 3, 2/7; cluster 4, 7/7; P<0.05). Oxytetracycline is widely used to treat PID and resistance to oxytetracycline also differed amongst the *E. coli* (cluster 1, 0/5; cluster 2, 0/7; cluster 3, 2/7; cluster 4, 7/7; P<0.05).

### *E. coli* or LPS Infused into the Uterus of Mice Causes PID or Endometritis

**Figure 6:**
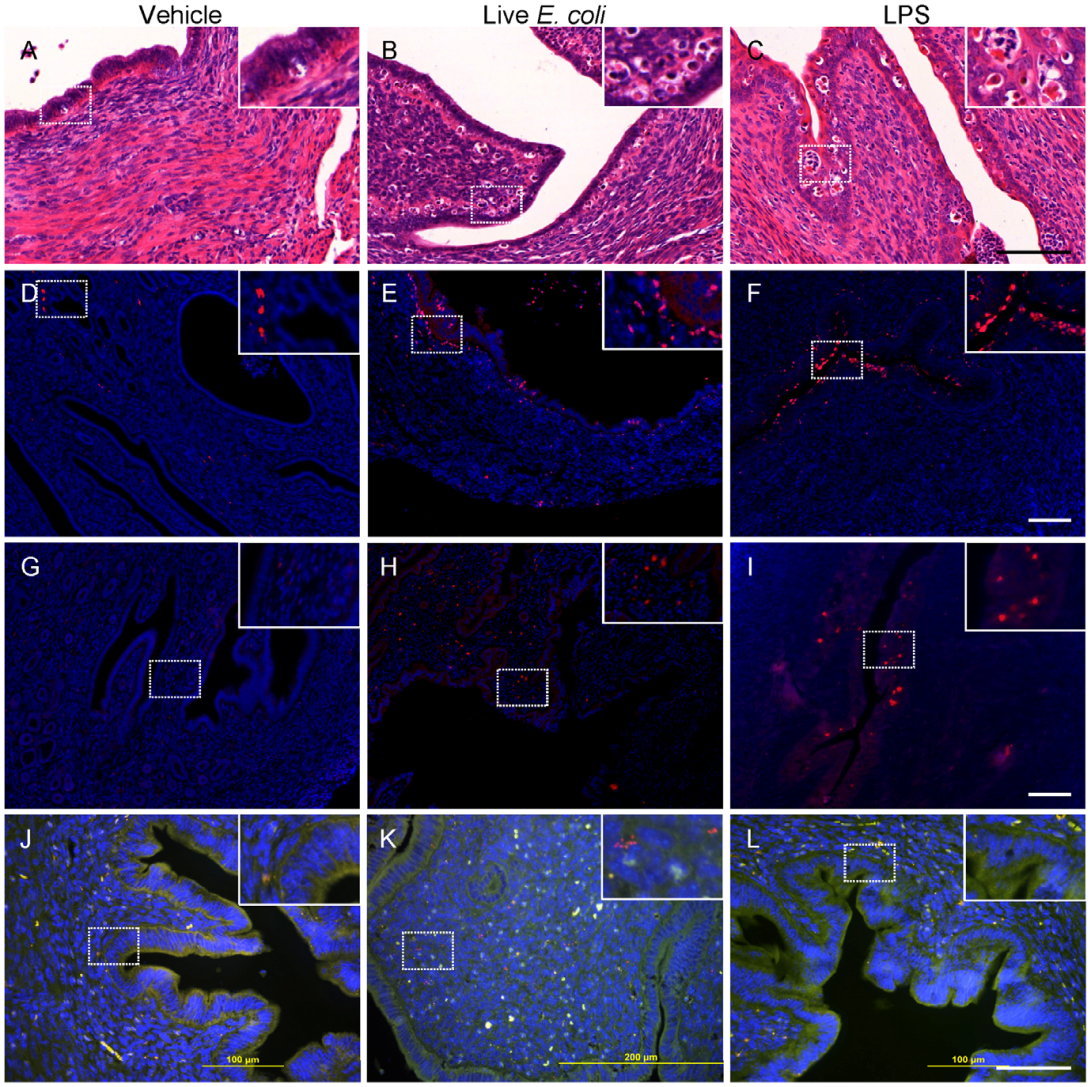
Uterine-derived *E. coli* cause PID in mice. Mice were infused with vehicle, live bacteria or LPS for 24 h and the uterus collected and processed for histology. Inflammation in the endometrium of animals treated with bacteria or LPS is evident in H&E sections (A–C) and following immunohistochemical staining for neutrophils (D–F) or macrophages (G–I). Bacteria invasion of the uterus is evident using FISH for mice infused with *E. coli* into the uterine lumen (J–L).

Live bacteria and purified LPS from MLST cluster 1 and 4 *E. coli* were infused into the uterine lumen of C57BL6 mice. Within 24 h of infusion 5/5 mice administered the cluster 4 O73:H16 bacteria showed signs of toxaemia (cold extremities, reduced appetite, inactivity), one animal had a uterus distended with purulent material and one animal died. No clinical signs were observed in animals administered cluster 1 O(84,172):H+ bacteria (n = 5), LPS from either cluster (n = 5 per cluster) or vehicle (n = 5). The endometrium from the mice infused with live bacteria or LPS was inflamed with evidence of neutrophil accumulation (Fig. 6A–C). Immunhistochemistry confirmed the location of neutrophils (Fig. 6D–F) and macrophages (Fig. 6G–I) with the immune cells localised to the epithelium and lumen in mice infused with LPS but more widespread in the endometrium of mice infused with live bacteria. *E. coli* were detectable by fluorescence in situ hybridization (FISH) colonising the endometrium only in animals infused with bacteria, and could be seen invading throughout the endometrium and myometrium (Fig. 6J–L).

### LPS from Uterine *E. coli* Provokes Endometrial Cell Inflammation via TLR4

**Figure 7:**
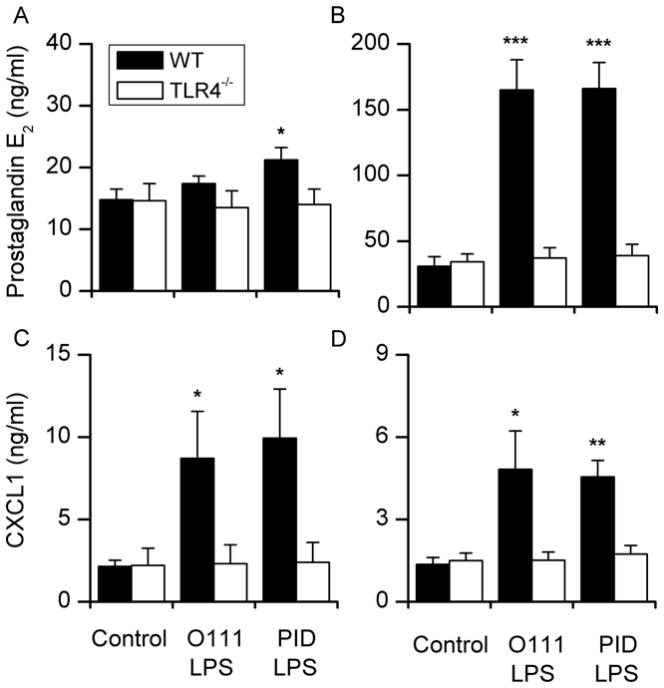
Endometrial cells respond to LPS and the response is TLR4 dependent. Prostaglandin E_2_ and CXCL1 concentrations accumulated over 24 h in the medium of endometrial epithelial (A, C) and stromal cells (B, D) isolated from C57BL/6 wild type (WT) or TLR4^−/−^ mice. Cells were treated with control media or media containing 1 µg/ml LPS purified from *E. coli* O111:B4 (O111 LPS) or from bacteria isolated from the uterus classified into MLST cluster 4 (PID LPS; n = 2 isolates). Prostaglandin E_2_ and CXCL1 concentrations were determined by RIA and ELISA, respectively. Data are presented as the mean + SEM for three experiments. Values differ significantly between WT and TLR4^−/−^, * P<0.05, ** P<0.01, *** P<0.001.

The key receptor for LPS on professional immune cells is TLR4, leading to activation of immune and inflammatory pathways [17]. To test the role of TLR4 in the endometrium, epithelial and stromal cells from C57Bl6 wild type or TLR4^−/−^ mice were challenged with LPS purified from MLST cluster 4 *E. coli*, using commercially purified LPS as a positive control. As for the bovine cells, the response to LPS was tested by measuring the accumulation in the medium of prostaglandin PGE and a chemokine similar to IL-8, chemokine (C-X-C motif) ligand 1 (CXCL1; also known as keratinocyte-derived chemokine, KC). Epithelial and stromal cells from wild type but not TLR4^−/−^ mice secreted PGE and CXCL1 in response to LPS (Fig. 7).

## Discussion

Gram-negative infections of the upper female genital tract are an important cause of PID and infertility in humans and animals [2], [9]. The present study focussed on *E. coli* infection after parturition, which paves the way for other pathogens, causes PID and leads to infertility [7], [9], [18]. The expectation was that the bacteria that cause PID were multiple genetically diverse opportunist pathogens of the endometrium. However in the present study, clonal groups of *E. coli* identified by Triplex PCR, RAPD and MLST were isolated from the endometrium of postpartum animals. The *E. coli* isolated from the uterus lacked many common virulence genes associated with DEC or ExPEC bacteria. The clonal *E. coli* associated with PID were more adherent and invasive for endometrial epithelial and stromal cells than clonal bacteria isolated from clinically unaffected animals. The bacteria or the LPS caused PID or endometritis in mice when infused into the uterus *in vivo*. Furthermore, LPS purified from the *E. coli* associated with PID provoked inflammatory responses by bovine endometrial cells *in vitro*. Murine endometrial cells also responded to LPS via a TLR4 dependent pathway. Taken together, the data provide evidence for specific strains of endometrial pathogenic *E. coli* that cause PID, which were designated as EnPEC. The implications of the findings are that development of vaccines or biological therapeutics for PID should specifically target EnPEC rather than other *E. coli* strains.

The *E. coli* isolated from the uterus of postpartum animals were mainly from Triplex-PCR groups A, B1 and D, with only 3 isolates from the B2 category. Group A and B1 *E. coli* are usually considered commensal in the intestine and are commonly shed into the feces of healthy animals [11], [12], [28]. On the other hand, ExPEC preferentially belong to group B2 and D [28] and *E. coli* that cause mastitis strains are usually group A or D [23]. Although the distribution of phylogroups in the present study was similar to that for *E. coli* isolated from feces, fecal *E. coli* have a wide genetic diversity between animals and over time [29]. Indeed, the isolates from animals with PID were predominantly in the B1 and A groups and more likely to be collected during the first two weeks post partum, whereas *E. coli* from the uterus of unaffected animals were distributed more equally between the A, B1 and D groups, and throughout the first four weeks after parturition. The RAPD analysis revealed that multiple isolates within a phylogroup were similar in overall genotype, and this refined subgroup of *E.coli* strains was further characterized by MLST and serotyping. A cluster of *E. coli* was identified by MLST that was only isolated from unaffected animals (cluster 1) and three clusters of bacteria were associated with PID (clusters 2 to 4). In addition, the uterine derived bacteria clustered apart from 11 reference strains of *E. coli* evaluated in the MLST dendrogram, including DEC, ExPEC and a bovine mastitis strain. Strains from PID and unaffected animals had diverse serogroups. However, when analyzed in concert with MLST it was apparent that all of the *E. coli* strains in cluster 1 shared the same serotype O(84,172:H+, represented by uncharacterized MLST st7 and clonal groups, which differ from diarheagenic *E. coli* or isolates from the gut of healthy animals [11], [12], [16]. The strains in cluster 4 derived solely from animals with PID were all serotype O73:H16 st7 461 clonal group 41. The *E. coli* strain O73:H16 has been previously isolated from cattle and is not considered pathogenic [30], although shigatoxin containing *E.coli* O73:H16 has been associated with bloody diarrhea and the prototypical *E.coli* in clonal group 41 (TW08574) is also a shiagtoxin producing strain. However the 073:H16 strains associated with PID in the present study are negative for shigatoxin genes. Given the clear segregation of *E. coli* clusters from unaffected animals and those with PID by MLST (98–100% bootstrap values for each cluster), we therefore explored how the clusters of uterine *E. coli* associated with PID differed from the bacteria collected from the endometrium of unaffected animals.

Essential pathogenicity traits include adhesion to host cells [14], motility mediated by flagella (identified by the H serogroup) [13], and toxins such as LPS (identified by the O serogroup) [16]. The MLST cluster 2, 3 or 4 bacteria were more adherent and invasive for endometrial cells than bacteria from cluster 1. FimH adhesion of type 1 pili is an important adhesion and invasion factor for UPEC [20]. Adhesion of the uterine *E. coli* to the primary endometrial cells in the present study was also at least in part mediated by FimH because adhesion could be reduced by D-Mannose. Infusion of carbohydrates that bind Type 1 pili may be useful for prevention of PID [31]. It was also interesting to note that pre-treatment of host cells with steroids modulated bacterial adhesion because endometrial cells are exquisitely sensitive to ovarian steroids, which control their biology as well as influencing the risk of PID [19], [22]. Endometrial cell invasion by bacteria increased over time but was not associated with cell death, at least up to 4 h of incubation. Invasion was at least in part associated with host cell cytoskeleton as inhibitors of microtubules and microfilaments markedly reduced bacterial invasion, similar to previous reports for pathogenic *E.coli* in other other tissues [23]. Indeed, this pattern of inhibition is similar to that of enteroinvasive and meningitis associated *E.coli* that coapt cytoskeletal elements to gain entry to cells, and differs from *Salmonella typhimurium* which is not inhibited by colchicine. In summary, the adhesive and invasive *E. coli* associated with PID provide evidence for specific strains of endometrial pathogenic *E. coli* (EnPEC) that cause PID.

A limitation of the present study was that it occurred in a single dairy herd. However, identifying the *E. coli* that are pathogenic in the endometrium and cause PID is important. The consequences of uterine infection include reduced milk yield and infertility, which costs the USA dairy industry $650 million per annum [9]. Indeed, our study is not alone and there is emerging evidence from Europe and the USA that *E. coli* triplex B1 strains are prevalent in PID across several locations [32]. However, the evolutionary background of EnPEC is not clear. One possibility is that the endometrial pathogens may have originated and evolved from intestinal *E. coli*, as fecal contamination of the vulva and vagina is common. The relationship between EnPEC and DEC or ExPEC requires further exploration before firm conclusions can be drawn.

There was little evidence that the uterine *E. coli* isolates possessed the common pathogenicity genes commonly associated with adhesion, invasion and virulence of DEC or ExPEC although the 17 genes tested only represent a small proportion of the available total [14], [26]. It is noteworthy that the *fyuA* gene was found in EnPEC but not *E. coli* from the uterus of clinically unaffected animals. The *fyuA* gene encodes the outer membrane protein ferric yersiniabactin uptake that is important for iron uptake and for biofilm formation in UPEC [27], and the scavenging of iron by bacteria in the endometrium may warrant further investigation. The identification of novel pathogenicity genes in EnPEC for the endometrium is likely best accomplished by genome sequencing.

Cells recognise LPS via the TLR4/MD-2/CD14 complex and much of the pathology associated with Gram-negative bacteria is associated with the binding of LPS to TLR4 [17]. Endometrial cells challenged with LPS preferentially secrete PGE [18], [19] and the chemokine IL-8, which attracts neutrophils to the endometrium [24]. In the present study, LPS purified from MLST cluster 4 bacteria stimulated the greatest accumulation of PGE or IL-8 in bovine endometrial cells. Differences in the the cellular inflammatory response between LPS from different *E. coli* may be associated with structural differences between the LPS of different bacterial strains [33]; or differences in other virulence mechanism that result in exposure of the endometrium to LPS. Endometrial cells isolated from wild type mice also secreted PGE and the chemokine CXCL1, which is the murine chemokine similar to IL-8, in response to LPS from EnPEC but not more than the ultrapure LPS. The cellular response to LPS was abrogated in endometrial cells purified from TLR4^−/−^ mice. These data confirm the important role that TLR4 has in binding LPS during the generation of the innate immune response [17]. Furthermore, it is clear that TLR4 on endometrial epithelial and stromal cells plays an important role in the response to bacterial infection of the uterus and the development of PID [18], [19], [34].

An important observation from the present study was that the EnPEC could cause PID in mice and that the clinical disease was more severe when cluster 4 rather than cluster 1 bacteria were infused into the uterine lumen. There was histological evidence of neutrophil and macrophage accumulation in the endometrium with bacteria or LPS infused into the uterine lumen but the response was mainly mucosal for LPS rather than across the stroma for the live bacteria. Furthermore, the EnPEC also colonised the endometrium and invaded throughout the uterine wall. A mouse model of PID may be useful to study host-pathogen interactions and the mechanisms of infection and immunity in the endometrium. The present work has most relevance for PID in cattle rather than humans because the bacteria infecting the upper female genital tract differ between species [1], [3], [6], [7]. However, an *in vivo* model for the endometrium *per se* is important because mucosal immunity mechanisms in the endometrium appear to be different from the other commonly studied mucosa such as the intestinal or respiratory tracts, and are closely regulated by ovarian steroids [35], [36].

In conclusion, the present study counters the previous hypothesis that bacteria causing PID are genetically diverse isolates from the feces or environment that randomly infect the reproductive tract. Here we have identified for the first time specific strains and clonal groups of *E. coli* that posses a pathogenic potential for causing PID in cattle, which we call endometrial pathogenic *E. coli* (EnPEC). The clusters of *E. coli* identified using MLST differed from reference strains of *E. coli*, including DEC, ExPEC and a bovine mastitis strain, but further genotyping is required to understand their origins. The EnPEC did not posses invasins, adhesins and virulence genes typical of DEC- or ExPEC, except for *fyuA*, which is associated with iron scavenging. The *E. coli* strains associated with PID were most adherent and invasive for endometrial cells. These *E. coli* stimulated a host cell immune response, which was at least in part mediated by LPS binding to TLR4 on endometrial cells. The EnPEC were also used to establish a murine model of PID but whether EnPEC cause disease in humans requires investigation. The implications of the findings from the present study provide a paradigm shift for development of vaccines or biological therapeutics for PID, which should specifically target EnPEC rather than other strains of *E. coli*.

## Materials and Methods

### Ethics Statement

All procedures were conducted under the UK Animal Scientific Procedures Act (1986) with the approval of the UK Government Home Office, the Royal Veterinary College Local Ethical Review Committee and Swansea University Ethical Review Process committee.

### Collection of *E. coli* from the Uterus

All procedures were conducted under the UK Animal Scientific Procedures Act (1986) with the approval of the UK Government Home Office and the Royal Veterinary College Local Ethical Review Committee. All postpartum Holstein-Friesian cows in the Royal Veterinary College dairy herd (median parity 3; range: 1 to 7) were maintained under standard conditions and examined as described during a one-year period [6]. Animals with non-uterine bacterial infections were excluded. Uterine disease was evaluated as described previously with animals categorised as having PID if they had pus in the mucus discharge from the cervix [6], [9]. To isolate bacteria from the uterus, a transcervical guarded swab was collected from the uterine body of clinically unaffected and diseased animals 7, 14, 21 and 28 days after parturition [6]. Swabs were processed for microbiology as described previously [6]. Briefly, each swab was transferred to a bijou bottle containing Stuart transport medium (Unipath, Basingstoke) and was cultured within 1 h of collection at the on-site bacteriology laboratory. Swabs were cultured aerobically and anaerobically on pre-equilibrated sheep blood agar (Oxoid, UK), and aerobically on MacConkey agar (Oxoid, UK). Identification of bacteria was based on the characteristics of the colony, Gram stain, morphology, haemolysis, biochemical profile (API systems, BioMérieux, Basingstoke) and other standard tests as previously described [37]. All *E. coli* isolates were sub-cultured and stored at −80°C in 20% glycerol, 10% skimmed milk.

### Bacterial Phylogeny

The triplex PCR [38] was used to determine phylogenetic group (A, B1, B2, or D) of the isolates. The genetic diversity of *E. coli* strains were evaluated by randomly amplified polymorphic DNA (RAPD)-PCR with informative primers 1254, 1281, and 1283 as previously described [25], [39]. *E. coli* isolates were serotyped (O and H antigens) at the *E. coli* serotyping Reference Center at Pennsylvania State University (University Park, PA).

Multilocus sequence typing (MLST) for seven loci (*aspC, clpX, fadD, icdA, lysP, mdh, uidA*) was performed according to the established MLST protocols for *E. coli* (http://www.shigatox.net/ecmlst/protocols/index.html; *Ec*MLST, Michigan State University). Column purified PCR amplicons were sequenced at the Cornell University BioResource Center, using forward and reverse PCR primers and an ABI 3700 automated DNA sequencer and ABI PRISM BigDye Terminator Sequencing kits with AmpliTaq DNA Polymerase (Applied Biosystems, Foster City, CA, USA). DNA sequences obtained with both forward and reverse primers were proofread, and then assembled in SeqMan (DNAStar, Madison, WI, USA). Sequences were aligned using the Clustal-W algorithm. A neighbor-joining tree with Jukes Cantor corrections was constructed in MEGA 4 software. Bootstrap values were calculated from 1000 replicate analyses. The following *E. Coli* reference strains from different pathogroups were included in the MLST tree: avian pathogenic *E. coli* (APEC) 110 (TW08895, source: bird), enteropathogenic *E. coli* (EPEC) C189-54 (TW06578, source: human), enteroadherent *E. coli* (EAEC) Peru H46-2 (TW08990, source: human), enteroinvasive *E. coli* (EIEC) 1885-77 (TW01095, source: human), enterohaemorrhagic *E. coli* (EHEC) O157:H7 86-24 (TW00116, source: human), EHEC DEC8c (TW01378, source: cow), Shiga toxin-producing *E. coli* (STEC) 537/89 (TW07863, source: cow), STEC BCL69 (TW05145, source: cow), STEC S102-9 (TW01496, source: cow), UPEC CFT073 (TW08018, source: human), *E. Coli* K12 MG1655 (TW08017) and mastitis *E. Coli* strain ECA-B. Control sequences were obtained from http://www.shigatox.net/ecmlst/cgi-bin/strainquery and the sequence of ECA-B was provided by Dr. Kenneth Simpson. Allele, st7 and clonal group were determined using the *Ec*MLST web-based software (http://www.shigatox.net/ecmlst/cgi-bin/mlbquery).

### Bovine Primary Endometrial Cell Isolation and Culture

Primary endometrial epithelial and stromal cells were isolated and cultured as described previously [18]. Briefly, bovine uteri were collected from post-pubertal non-pregnant animals with no evidence of genital disease or microbial infection at a local abattoir and kept on ice until further processing in the laboratory. The stage of the reproductive cycle was determined by observation of ovarian morphology and genital tracts with an ovarian Stage II corpus luteum were selected for endometrial culture [40]. The endometrium from the horn ipsilateral to the corpus luteum was cut into strips and placed into PBS (Sigma, Poole, UK) supplemented with 50 IU/ml of penicillin, 50 µg/ml of streptomycin and 2.5 µg/ml of amphotericin B (Sigma). The endometrial strips were then cut into smaller pieces and placed into HBSS as previously described [41]. Briefly, tissue was digested in 25 ml sterile digestive solution, made by dissolving 50 mg trypsin III (Roche, Welwyn, UK), 50 mg collagenase II (Sigma), 100 mg BSA (Sigma) and 10 mg DNase I (Sigma) in 100 ml HBSS. Following 1.5 h incubation in a shaking water bath at 37°C, the cell suspension was filtered through a 40 µm mesh (Fisher Scientific, Loughborough, UK) to remove undigested material and the filtrate was resuspended in washing medium, comprising of HBSS with 10% fetal bovine serum (FBS, Sigma). The suspension was centrifuged at 700 x *g* for 7 min in washing medium twice before the cells were re-suspended in RPMI-1640 medium containing 10% FBS, 50 IU/ml of penicillin, 50 µg/ml of streptomycin and 2.5 µg/ml of amphotericin B. The cells were plated at a density of 1×10^5^ cells/ml in 24-well plates (Helena Bioscience, Gateshead, UK). To obtain separate stromal and epithelial cell populations, the cell suspension was removed 18 h after plating, which allowed selective attachment of stromal cells [41]. The removed cell suspension was then re-plated and incubated allowing epithelial cells to adhere [42]. Stromal and epithelial cell populations were distinguished by cell morphology as previously described [41]. The culture media was changed every 48 h until the cells reached confluence. Cell cultures were maintained at 37°C, 5% CO_2_ in air, in a humidified incubator.

### Bacterial Adhesion and Invasion Assays for Endometrial Cells

Primary bovine endometrial epithelial and stromal cells were cultured in 24-well plates (TPP, Trasadingen, Switzerland) until confluent and the number of cells was measured using a Coulter counter. Bacteria were grown for 24 h in LB broth (Sigma) at 37°C, harvested by centrifugation at 8,000 x g for 10 min, washed twice in PBS, and re-suspended in 10 ml PBS. The number of CFU/ml was determined by plate counts on LB agar plates. For adhesion experiments, bacteria were added to mammalian cells in fresh medium at 10 M.O.I. and incubated for 1 h at 37°C. Medium was removed from each well and the cells washed four times with PBS to remove non-adherent bacteria before lysis of cells with PBS, 1% Triton X-100, 0.1% SDS, and the number of CFU/ml determined by plate counts. To explore the role of FimH, adhesion experiments were done in the presence of 2.5% D-Mannose. To explore the role of ovarian steroids, endometrial cells were grown in control culture medium, or in medium containing progesterone at luteal phase concentrations or estradiol at ovarian follicular phase concentrations, for 48 h before measuring bacterial adhesion. For invasion assays 10 M.O.I. bacteria were added to endometrial cells for 1, 2, 3 or 4 h; supernatant removed; the cells washed twice with PBS; the cells were then incubated for 1.5 h with medium containing 50 µg/ml gentamicin to kill extracellular bacteria. The medium was then removed from the cells, which were washed three times with PBS, lysed and CFU/ml determined. To explore the mechanism of invasion, gentamicin protection assays were also performed with endometrial cells that had been incubated for 1 h in control medium or in medium with 1 µg/ml cytochalasin D or 5 µg/ml colchicine before addition of 10 M.O.I. bacteria. To confirm the presence of FimH, agglutination of *S. cerevisiae* was performed as previously described [43]. Experiments were performed in duplicate on at least 4 separate occasions, using at least 4 strains of *E. coli* randomly selected from each MLST cluster. The *E. coli* in cluster 1 were from unaffected animals whereas isolates tested from clusters 2 to 4 were from animals with PID.

### LPS Purification

Bacteria were grown in 150 ml Brain Heart Infusion broth (Oxoid) at 37°C overnight with shaking (200 rpm) and then centrifuged at 5000 x g for 10 min. The supernatant discarded and bacteria washed in pure water for 3 cycles. The bacterial pellet was suspended in 100 ml 10 mM Tris 10 mM EDTA buffer, pH 8.0 to disrupt the outer membrane. An equal volume of 80% w/w phenol was added and mixture emulsified at 67°C for 60 min in a shaking water bath. The phases were separated at 4°C overnight and the aqueous phase collected and re-extracted with a second round of hot phenol. The resulting aqueous phase was extensively dialysed against water for 24 h, changing the water every 2 h. The dialysis solution was centrifuged for 2 h at 100,000 x g (Beckman L8-70M ultracentrifuge), supernatant discarded and the LPS pellet suspended in pure water. Concentration of LPS was determined by Limulus Amebocyte Lysate assay (QCL-1000; Lonza, Basel, Switzerland).

### Endometrial Cell Response to LPS

Lipopolysaccharides purified from 3 uterine *E. coli* isolates per MLST cluster were used at a concentration of 1 µg/ml in culture media to challenge endometrial epithelial or stromal cells in 24-well plates, using untreated media or media containing 1 µg/ml of commercially ultrapurified LPS from *E. coli* O111:B4 as negative and positive controls, respectively. The LPS derived from *E. coli* in cluster 1 were from unaffected animals whereas isolates from clusters 2 to 4 were from animals with PID. Preliminary experiments with bacteria in cluster 3 found no difference between the strains from diseased and unaffected animals Supernatants were collected after 24 h and analysed for steady-state PGE and IL-8 concentrations. Supernatants were analysed for steady-state PGE concentrations by radio-immunoassay (RIA) as previously described [19]. Briefly, samples were diluted in 0.05 M Tris buffer containing 0.1% gelatin and 0.01% sodium azide. Standards and tritiated tracers were purchased from Sigma and Amersham International PLC (Amersham, UK), respectively. The antisera were a generous gift from Professor N.L. Poyser (University of Edinburgh, UK) and their cross-reactivity have been reported [44]. The limit of detection for PGE was 2 pg/tube and intra- and inter-assay coefficients of variation were 4.4% and 7.8%, respectively. Supernatant IL-8 concentrations were determined with a commercially available human IL-8 ELISA kit (R&D Systems, Inc., Minneapolis, MN), where the antibody pairs have been previously shown to cross-react with bovine IL-8, as previously described [45].

### Immunocytochemistry

Primary bovine endometrial epithelial or stromal cells were grown to 80% confluence in 8-well chamber slides (Lab-tek, Nalgene Nunc International, Naperville, IL, USA). Bacteria were added at 10 M.O.I. to the cells and incubated for 1 or 4 h at 37°C, with SYTO® 9 green fluorescent nucleic acid stain added 30 min before the supernatants were carefully removed and the cells washed twice in sterile PBS (Lonza, Verviers, Belgium). The cells were then fixed for 10 min in 4% PFA (Sigma), and washed three times in PBS 0.1% Triton for 3 min. and mounted with 4′,6-diamidino-2-phenylindole (Vectashield with DAPI; Vector Laboratories Inc., Burlington, CA, USA). Slides were examined using a confocal microscope (Zeiss 510 LSM, Jena, Germany).

### Virulence Factors

*E. coli* isolates were screened by PCR for the presence of genes encoding virulence factors at the *E. coli* Reference Center, Pennsylavania State University, using primers that have been previously described [46] for K99 fimbrial subunit (Genbank accession number FJ864678), *E.coli* fimbrial adhesin subunit F1845 (Genbank M27725), *E.coli* CS31A fimbrial subunit precursor (Genbank M59905), heat-stable toxin, STa Shiga-like toxin types 1 and 2, *stx1* and *stx2*, cytotoxic necrotizing factors 1 and 2 (*cnf1* and *cnf2*), and intimin-*γ* (*eae*),. The presence of colV, *kpsMII*, *fyuA*, *ibeA, papC, afaB-afaC, sfaD-sfaE* and *focG* was determined by PCR as previously described [25], [47], [48].

### Antimicrobial Sensitivity

A selected sensitivity profile was determined for isolates of *E. coli* by disc diffusion as outlined in NCCLS Performance Standards except that Isosensitest medium was used in place of Mueller-Hinton. A single colony of each isolate cultured overnight on Isosensitest agar (Oxoid) was suspended in 1 ml of 100 mM phosphate buffered saline, pH 7.3. The suspension (approx. 2.×10^5^ CFU/ml) was spread over the surface of Isosensitest agar and antibiotic discs applied. Discs, obtained from Oxoid UK, were used at the following strengths: ampicillin, 10 µg; amoxicillin-clavulanic acid, 30 µg; oxytetracycline, 30 µg; ceftiofur, 30 µg; enrofloxacin, 5 µg; streptomycin 10 µg; sulphonamide-trimethoprim, 25 µg. Following incubation for 18 hours, cultures were examined and the diameter of each zone of inhibition was recorded in mm [49]. Data are reported for all the available isolates where the results were unequivocal (n = 90), which included all the *E. coli* strains from clusters 1 to 4.

### Mouse Disease Model

All procedures were conducted under the UK Animal Scientific Procedures Act (1986) with the approval of the UK Government Home Office and the Royal Veterinary College Local Ethical Review Committee. Female C57BL/6 (WT) mice were purchased from Charles River Laboratories (Margate, Kent, UK). Breeding colonies were maintained under standardized conditions, in a specific pathogen-free environment, with free access to water and a standard breeding or maintenance rodent diet as appropriate. Female C57BL/6 mice 6–8 weeks old were infused intra-uterine with 10^4^ CFU cluster 1 or 4 *E. coli*, 100 µg LPS from cluster 1 or 4 *E. coli*, or vehicle using a 27G needle inserted into the tip of the left uterine horn (n = 5 per treatment), which was visualised by a laparotomy incision under general anaesthesia. Mice were monitored every 2 h for signs of health until 24 h after infusion when the genital tract was collected, fixed overnight in 10% formalin, transferred to 70% ethanol and paraffin embedded (FFPE). The FFPE sections were cut at 6 µm, deparaffinised and stained using Haematoxylin and Eosin at the same time using the Leica Auto Stainer XL (Leica, New Jersey, USA). Images were examined by Mirax Scan and Mirax Viewer software (Zeiss, Jena, Germany). Sections were also immunostained with antibody against neutrophils (clone 7/4; AbD, Kidlington, UK) or macrophages (F4/80; AbD) using Alexafluor 555 secondary detection antibody (Invitrogen, Paisley, UK) with nuclei stained using DAPI (Vector Labs,Burlingame, CA, USA). Images were taken using a Zeiss upright microscope and an AxioCam, with scale bars added by the software (Axiovision, Zeiss). Fluorescence in situ hybridization (FISH) was performed on deparaffinized formalin fixed sections using a eubacterial probe (EUB3380Cy3) and antisense control probe (nonEUB338-FAM), and visualized on an Olympus BX-51 fluorescense microscope and DP70 camera as previously described [25].

### Murine Primary Endometrial Cell Isolation and Culture

Primary endometrial epithelial and stromal cells were isolated and cultured as described previously [50]. Each experiment used 6 to 8 C57BL/6 wild type purchased from Charles River Laboratories (Margate, Kent, UK) or TLR4^−/−^ mice on a C57BL/6 background, which were a kind gift from Prof. S. Akira (Osaka University, Japan). All procedures were conducted under the UK Animal Scientific Procedures Act (1986) with the approval of the UK Government Home Office and the Royal Veterinary College Local Ethical Review Committee. Experiments were repeated on three separate occasions. The cells were plated at a density of 5×10^5^ cells/ml in 24-well plates (Helena Bioscience, Gateshead, UK). Cell cultures were maintained at 37°C, 5% CO_2_ in air, in a humidified incubator. The culture media was changed after 48 h until and the cells treated with control media or 1 µg/ml LPS from *E. coli* O111:B4 or LPS from MLST cluster 4 uterine *E. coli* (n = 2 isolates). Supernatants were collected after 24 h and analysed for steady-state PGE and CXCL1 concentrations. Supernatants were analysed for steady-state PGE concentrations by radio-immunoassay (RIA) as described above. Supernatant CXCL1 concentrations were determined with a commercially available murine CXCL1 ELISA kit (R&D Systems, Inc., Minneapolis, MN, USA).

### Statistical Analysis

The proportions of animals in phylogenetic or disease groups were compared by Chi-square analysis. Remaining data were compared by GLM univariate analysis.

## Supporting Information

Figure S1 Genetic diversity of *E. coli* strains. *E. coli* were identified by Random Amplification of Polymorphic DNA (RAPD) with RAPD primers 1254 and 1283 using genomic DNA extracted from *E. coli* strains from clinically unaffected postpartum animals and animals with pelvic inflammatory disease (PID). The figure shows a representative result for the indicated RAPD genotypes; the first unlabelled lane is the negative control; the last unlabelled lane for each primer is the DNA ladder.
(4.39 MB TIF)

Figure S2 The number of animals per month (Jan to Dec; month 1 to 12) which yielded *E. coli* isolates that were categorised by MLST into clusters 1 to 4.
(3.05 MB TIF)

Figure S3 Uterine *E. coli* express Type 1 fimbriae. Aggregation of yeast by *E. coli* isolates from MLST clusters 1 to 4. Bacteria were incubated with *Saccharomyces cerevisiae* yeast and absorbance at 600 nm measured every 15 min for 1 h; experiments were repeated on 3 separate occasions with 3 isolates from each cluster. Data are reported as optical density (OD) readings.
(1.49 MB TIF)

Figure S4 Uterine *E. coli* antimicrobial sensitivity. Proportion of *E. coli* isolates from the bovine uterine lumen categorised in Triplex phylogroup A (n = 31), B1 (n = 41) or D (n = 18), that were sensitive to ampicillin (AMP), oxytetracycline (OT), streptomycin (Strep) and sulphonamide/trimethoprim (SxT), enrofloxacin (ENF), ampicillin and clavulanic acid (AMP/CLAV), or ceftiofur (CEF).
(3.05 MB TIF)

Table S1 Details of Triplex PCR group, serotype, MLST and clonal group (ST7:CG), bacterial class (NT, not typed; STEC, Shiga toxin-producing *E. coli*; ECOR-A, *E. coli* group A) and analysis of virulence genes by PCR (as defined in Material and Methods) for uterine *E. coli* isolates in RAPD groups B1-4, B1-1, A4 and D5 collected from the uterus of postpartum cattle with pelvic inflammatory disease (PID, 1) or from unaffected animals (0).
(0.23 MB DOC)
