# Supplementary figures and images for: Specific Strains of Escherichia coli Are Pathogenic for the Endometrium of Cattle and Cause Pelvic Inflammatory Disease in Cattle and Mice
Source: PLoS One. 2010 Feb 12;5(2):e9192. doi: 10.1371/journal.pone.0009192 (PMC2820550; doi:10.1371/journal.pone.0009192)

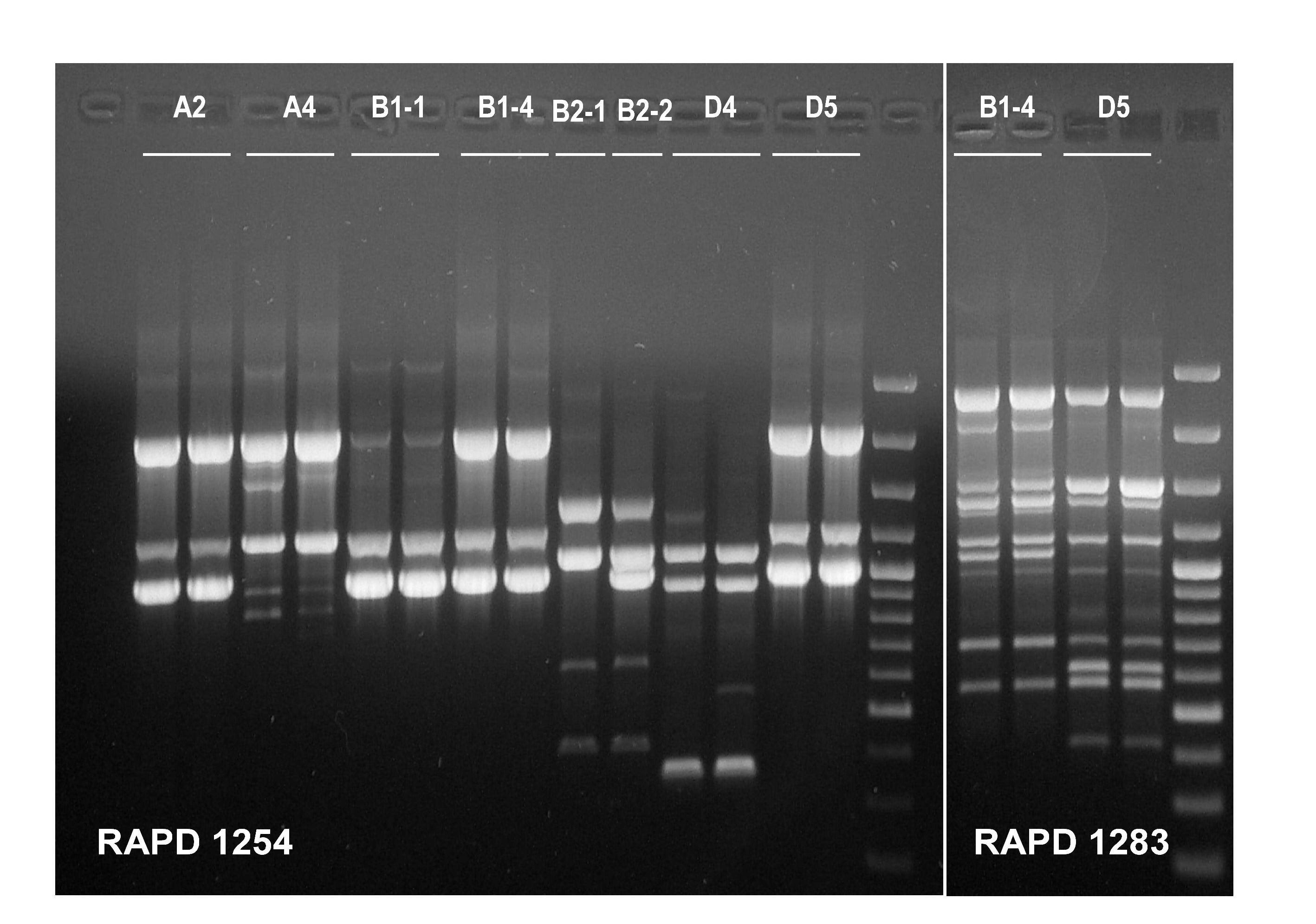

Supplement: Figure S1 — Genetic diversity of E. coli strains. E. coli were identified by Random Amplification of Polymorphic DNA (RAPD) with RAPD primers 1254 and 1283 using genomic DNA extracted from E. coli strains from clinically unaffected postpartum animals and animals with pelvic inflammatory disease (PID). The figure shows a representative result for the indicated RAPD genotypes; the first unlabelled lane is the negative control; the last unlabelled lane for each primer is the DNA ladder. (4.39 MB TIF) [file pone.0009192.s001.tif]
