# Supplementary material for: Specific Strains of Escherichia coli Are Pathogenic for the Endometrium of Cattle and Cause Pelvic Inflammatory Disease in Cattle and Mice
Source: PLoS One. 2010 Feb 12;5(2):e9192. doi: 10.1371/journal.pone.0009192 (PMC2820550; doi:10.1371/journal.pone.0009192)
Supplement: Table S1 — Details of Triplex PCR group, serotype, MLST and clonal group (ST7:CG), bacterial class (NT, not typed; STEC, Shiga toxin-producing E. coli; ECOR-A, E. coli group A) and analysis of virulence genes by PCR (as defined in Material and Methods) for uterine E. coli isolates in RAPD groups B1-4, B1-1, A4 and D5 collected from the uterus of postpartum cattle with pelvic inflammatory disease (PID, 1) or from unaffected animals (0). (0.23 MB DOC) [file pone.0009192.s005.doc]

**Table S1**

Details of Triplex PCR group, serotype, MLST and clonal group (ST7:CG), bacterial class (NT, not typed; STEC, Shiga toxin-producing *E. coli*; ECOR-A, *E. coli* group A) and analysis of virulence genes by PCR (as defined in Material and Methods) for uterine *E. coli* isolates in RAPD groups B1-4, B1-1, A4 and D5 collected from the uterus of postpartum cattle with pelvic inflammatory disease (PID, 1) or from unaffected animals (0).

| **Isolate** | **Triplex** | **RAPD group** | **PID** | **Serotype** | **aspC:clpX:fadD:icdA:lysP:mdh:uidA** | **ST7:CG** | **Class** | **STa** | **STX1** | **STX2** | **CNF1** | **CNF2** | **EAE** | **K99** | **CS31A** | **F1845** | **colV** | **kpsMII** | **fyuA** | **afaBC** | **focG** | **ibeA** | **papC** | **sfaDE** |
| --- | --- | --- | --- | --- | --- | --- | --- | --- | --- | --- | --- | --- | --- | --- | --- | --- | --- | --- | --- | --- | --- | --- | --- | --- |
| ms8 | B1 | B1-4 | 0 | O-:H+ | 5:6:2:24:1:13:23 | 128:56 | NT-22 | - | - | - | - | - | - | - | - | - | - | - | - | - | - | - | - | - |
| ms14 | B1 | B1-4 | 0 | O-:H+ | 5:6:2:24:1:13:23 | 128:56 | NT-22 | - | - | - | - | - | - | - | - | - | - | - | - | - | - | - | - | - |
| ms462 | B1 | B1-4 | 1 | O-:H2 | 7:5:1:4:1:8:1 | *:0 | Undefined | - | - | - | - | - | - | - | - | - | - | - | - | - | - | - | - | - |
| ms470 | B1 | B1-4 | 1 | **O73:H16** | 5:5:2:30:1:8:1 | 461:41 | STEC | - | - | - | - | - | - | - | - | - | - | - | + | - | - | - | - | - |
| ms277 | B1 | B1-4 | 1 | O73:H16 | 5:5:2:30:1:8:1 | 461:41 | STEC | - | - | - | - | - | - | - | - | - | - | - | + | - | - | - | - | - |
| ms360 | B1 | B1-4 | 1 | **O73:H16** | 5:5:2:30:1:8:1 | 461:41 | STEC | - | - | - | - | - | - | - | - | - | - | - | + | - | - | - | - | - |
| ms273 | B1 | B1-4 | 1 | **O73:H16** | 5:5:2:30:1:8:1 | 461:41 | STEC | - | - | - | - | - | - | - | - | - | - | - | + | - | - | - | - | - |
| ms482 | B1 | B1-4 | 1 | O73:H16 | 5:5:2:30:1:8:1 | 461:41 | STEC | - | - | - | - | - | - | - | - | - | - | - | + | - | - | - | - | - |
| ms499 | B1 | B1-4 | 1 | O73:H16 | 5:5:2:30:1:8:1 | 461:41 | STEC | - | - | - | - | - | - | - | - | - | - | - | + | - | - | - | - | - |
| ms469 | B1 | B1-4 | 1 | O73:H16 | 5:5:2:30:1:8:1 | 461:41 | STEC | - | - | - | - | - | - | - | - | - | - | - | + | - | - | - | - | - |
| ms304 | B1 | B1-4 | 1 | O149:H2 | 5:*:2:2:1:8:* | *:0 | Undefined | - | - | - | - | - | - | - | - | - | - | - | - | - | - | - | - | - |
| ms468 | B1 | B1-4 | 1 | OX13:H2 | 7:5:2:4:1:8:1 | *:0 | Undefined | - | - | - | - | - | - | - | - | - | - | - | - | - | - | - | - | - |
| ms206 | B1 | B1-4 | 1 | O37:H10 | 5:5:79:39:1:5:1 | *:0 | Undefined | - | - | - | - | - | - | - | - | - | - | - | - | - | - | - | - | - |
| ms154 | B1 | B1-1 | 0 | O58:H27 | 5:6:2:25:1:8:1 | 425:30 | STEC | - | - | - | - | - | - | - | - | - | - | - | - | - | - | - | - | - |
| ms21 | B1 | B1-1 | 0 | O-:H+ | 5:6:2:24:1:13:23 | 128:56 | NT-22 | - | - | - | - | - | - | - | - | - | - | - | - | - | - | - | - | - |
| ms538 | B1 | B1-1 | 0 | O-:H8 | 5:5:13:2:1:13:23 | 243:31 | NT-5 | - | - | - | - | - | - | - | - | - | - | - | - | - | - | - | - | - |
| ms491 | B1 | B1-1 | 0 | O-:H30 | 5:6:2:25:1:8:1 | 425:30 | STEC | - | - | - | - | - | - | - | - | - | - | - | - | - | - | - | - | - |
| ms456 | B1 | B1-1 | 0 | O55:H21 | 5:6:2:25:1:8:1 | 425:30 | STEC | - | - | - | - | - | - | - | - | - | - | - | - | - | - | - | - | - |
| ms537 | B1 | B1-1 | 0 | O-:H7 | 7:5:2:2:1:13:1 | 140:31 | NT-5 | - | - | - | - | - | - | - | - | - | - | - | - | - | - | - | - | - |
| ms232 | B1 | B1-1 | 1 | O-:H7 | 5:5:12:*:1:13:23 | *:0 | Undefined | - | - | - | - | - | - | - | - | - | - | - | - | - | - | - | - | - |
| ms118 | B1 | B1-1 | 1 | O21w:H19 | 4:5:2:4:1:5:* | *:0 | Undefined | - | - | - | - | - | - | - | - | - | - | - | - | - | - | - | - | - |
| msV | B1 | B1-1 | 1 | O91:H7 | 5:58:5:2:1:13:84 | 374:56 | NT-22 | - | - | - | - | - | - | - | - | - | - | - | - | - | - | - | - | - |
| ms315 | B1 | B1-1 | 1 | OX13:H2 | 7:5:2:4:1:8:1 | *:0 | Undefined | - | - | - | - | - | - | - | - | - | - | - | - | - | - | - | - | - |
| ms318 | B1 | B1-1 | 1 | O8:H+ | 5:6:2:93:1:8:1 | 424:30 | STEC | - | - | - | - | - | - | - | - | - | - | - | + | - | - | - | - | - |
| ms361 | B1 | B1-1 | 1 | O91:H7 | 5:58:5:2:1:13:84 | 374:56 | NT-22 | - | - | - | - | - | - | - | - | - | - | - | - | - | - | - | - | - |
| ms373 | B1 | B1-1 | 1 | O58:H27 | 5:6:2:25:1:8:1 | 425:30 | STEC | - | - | - | - | - | - | - | - | - | - | - | - | - | - | - | - | - |
| ms374 | B1 | B1-1 | 1 | O8:H+ | 5:6:2:93:1:8:1 | 424:30 | STEC | - | - | - | - | - | - | - | - | - | - | - | + | - | - | - | - | - |
| ms320 | B1 | B1-1 | 1 | O6:H49 | 4:*:5:4:1:13:23 | *:0 | Undefined | - | - | - | - | - | - | - | - | - | - | - | - | - | - | - | - | - |
| ms467 | B1 | B1-1 | 1 | O54w:H2 | 7:5:13:2:1:13:1 | *:0 | Undefined | - | - | - | - | - | - | - | - | - | - | - | - | - | - | - | - | - |
| ms96 | B1 | B1-1 | 1 | O-:H8 | 5:5:2:4:1:53:63 | *:0 | Undefined | - | - | - | - | - | - | - | - | - | - | - | - | - | - | - | - | - |
| ms421 | B1 | B1-1 | 1 | O54w:H2 | 7:5:13:2:1:13:1 | *:0 | Undefined | - | - | - | - | - | - | - | - | - | - | - | - | - | - | - | - | - |
| ms459 | B1 | B1-1 | 1 | O21:H27 | 5:6:2:25:1:8:1 | 425:30 | STEC | - | - | - | - | - | - | - | - | - | - | - | - | - | - | - | - | - |
| ms209 | B1 | B1-1 | 1 | O21:H19 | 4:5:2:4:1:5:* | *:0 | Undefined | - | - | - | - | - | - | - | - | - | - | - | - | - | - | - | - | - |
| ms466 | A | A4 | 0 | O131w:H26 | 4:51:1:4:6:*:1 | *:0 | Undefined | - | - | - | - | - | - | - | - | - | + | - | - | - | - | - | - | - |
| ms446 | A | A4 | 0 | O-:H30 | 3:3:128:129:1:1:1 | 624:0 | Undefined | - | - | - | - | - | - | - | - | - | - | - | - | - | - | - | - | - |
| ms415 | A | A4 | 0 | O127:H(40 or 44) | 3:3:13:1:1:2:29 | 383:23 | ECOR-A | - | - | - | - | - | - | - | - | - | - | - | - | - | - | - | - | - |
| ms541 | A | A4 | 1 | O74:H39 | 3:3:1:1:1:1:1 | 171:23 | ECOR-A | - | - | - | - | - | - | - | - | - | - | - | + | - | - | - | - | - |
| ms546 | A | A4 | 1 | O74:H39 | 3:3:1:1:1:1:1 | 171:23 | ECOR-A | - | - | - | - | - | - | - | - | - | - | - | + | - | - | - | - | - |
| ms36 | A | A4 | 1 | O154:H(40 or 44) | *:3:1:1:1:1:29 | *:0 | Undefined | - | - | - | - | - | - | - | - | - | - | - | - | - | - | - | - | - |
| ms460 | A | A4 | 1 | O-:H32 | *:3:1:1:1:1:1 | *:0 | Undefined | - | - | - | - | - | - | - | - | - | - | - | - | - | - | - | - | - |
| ms367 | A | A4 | 1 | O-:H9 | 3:3:1:1:1:1:1 | 171:23 | ECOR-A | - | - | - | - | - | - | - | - | - | - | - | - | - | - | - | - | - |
| ms274 | A | A4 | 1 | O-:H4 | 4:12:13:1:1:1:134 | 397:78 | NT-44 | - | - | - | - | - | - | - | - | - | - | - | - | - | - | - | - | - |
| ms131 | A | A4 | 1 | O-:H+ | 3:3:1:1:1:1:1 | 171:23 | ECOR-A | - | - | - | - | - | - | - | - | - | - | - | - | - | - | - | - | - |
| ms75 | A | A4 | 1 | O-:H+ | 3:3:13:1:1:1:29 | 168:23 | ECOR-A | - | - | - | - | - | - | - | - | - | - | - | - | - | - | - | - | - |
| ms142 | A | A4 | 1 | O-:H10 | 3:3:1:15:1:1:1 | 169:23 | ECOR-A | - | - | - | - | - | - | - | - | - | - | - | - | - | - | - | - | - |
| ms133 | A | A4 | 1 | O-:H10 | 3:3:1:15:1:1:1 | 169:23 | ECOR-A | - | - | - | - | - | - | - | - | - | - | - | - | - | - | - | - | - |
| ms256 | A | A4 | 1 | O20:H12 | 10:12:2:12:1:12:12 | 86:13 | ETEC | - | - | - | - | - | - | - | - | - | - | - | + | - | - | - | - | - |
| ms197 | D | D5 | 0 | O(84,172):H+ | 11:31:*:69:*:*:* | *:0 | Undefined | - | - | - | - | - | - | - | - | - | - | - | - | - | - | - | - | - |
| ms151 | D | D5 | 0 | O(84,172):H+ | 11:31:*:69:*:*:* | *:0 | Undefined | - | - | - | - | - | - | - | - | - | - | - | - | - | - | - | - | - |
| ms25 | D | D5 | 0 | O(84,172):H+ | 11:31:*:69:*:*:* | *:0 | Undefined | - | - | - | - | - | - | - | - | - | - | - | - | - | - | - | - | - |
| ms28 | D | D5 | 0 | O(84,172):H+ | 11:31:*:69:*:*:* | *:0 | Undefined | - | - | - | - | - | - | - | - | - | - | - | - | - | - | - | - | - |
| ms211 | D | D5 | 0 | O(84,172):H+ | 11:31:*:69:*:*:* | *:0 | Undefined | - | - | - | - | - | - | - | - | - | - | - | - | - | - | - | - | - |
